# Economic analysis of costs with enteral and parenteral nutritional therapy according to disease and outcome

**DOI:** 10.1590/S1679-45082017GS4002

**Published:** 2017

**Authors:** Adriano Hyeda, Élide Sbardellotto Mariano da Costa

**Affiliations:** 1 Universidade Federal do Paraná, Curitiba, PR, Brazil.; 2 Instituto Superior de Administração e Economia, Fundação Getúlio Vargas, Curitiba, PR, Brazil.

**Keywords:** Malnutrition, Nutrition therapy, Costs and cost analysis, Health management, Health evaluation

## Abstract

**Objective:**

To conduct an economic analysis of enteral and parenteral diet costs according to the type of disease and outcome (survivors *versus* deaths).

**Methods:**

It is a cross-sectional, observational, retrospective study with a qualitative and quantitative design, based on analysis of hospital accounts from a healthcare insurance provider in the Southern region of Brazil.

**Results:**

We analyzed 301 hospital accounts of individuals who used enteral and parenteral diets. The total cost of the diet was 35.4% of hospital account total costs. The enteral modality accounted for 59.8% of total dietary costs. The major costs with diets were observed in hospitalizations related to infections, cancers and cerebro-cardiovascular diseases. The major costs with parenteral diet were with admissions related by cancers (64.52%) and dementia syndromes (46.17%). The highest ratio between total diet costs with the total of hospital account costs was in dementia syndromes (46.32%) and in cancers (41.2%). The individuals who died spent 51.26% of total of hospital account costs, being 32.81% in diet (47.45% of total diet value and 58.81% in parenteral modality).

**Conclusion:**

Enteral and parenteral nutritional therapies account for a significant part of the costs with hospitalized individuals, especially in cases of cancers and dementia syndromes. The costs of parenteral diets were higher in the group of patients who died.

## RESUMO

**Objetivo:**

Realizar uma análise econômica de custos da terapia nutricional enteral e parenteral, conforme o tipo de doença e o desfecho (sobreviventes *versus* óbitos).

**Métodos:**

Estudo transversal, observacional, retrospectivo, com estratégia qualitativa e quantitativa, a partir da análise de contas hospitalares de uma operadora de saúde da Região Sul do Brasil.

**Resultados:**

Foram analisadas 301 contas hospitalares de usuários que utilizaram dieta enteral e parenteral. O custo total com dieta foi de 35,4% do custo total das contas hospitalares. A modalidade enteral representou 59,8% do custo total em dieta. Os maiores custos com dieta foram observados em internações relacionadas a infecções, cânceres e doenças cérebro-cardiovasculares. Os maiores custos com dieta parenteral foram observados nas internações relacionadas aos cânceres (64,52%) e às síndromes demenciais (46,17%). A maior relação entre o custo total com dieta e o custo total da conta foi na síndrome demencial (46,32%) e no câncer (41,2%). Os usuários que foram a óbito consumiram 51,26% dos custos totais das contas, sendo 32,81% com dieta (47,45% do valor total com dieta e 58,81% do custo na modalidade parenteral).

**Conclusão:**

As terapias nutricionais enteral e parenteral representaram uma parte importante dos custos no tratamento de indivíduos hospitalizados, principalmente nos casos dos cânceres e nas síndromes demenciais. O custo com dieta parenteral foi maior no grupo de usuários que foram a óbito.

## INTRODUCTION

Malnutrition is observed in individuals that lack adequate quantities of calories, proteins, or other nutrients for the maintenance of their body functions. It occurs as a result of a complex interrelation between the underlying disease, the metabolic abnormalities related to the diseases, and reduced availability of nutrients (decreased ingestion, deficient absorption, increased losses, or combinations of all these factors).^1-3^ Nutritional therapy (NT) has the objective of providing the necessary quantity of nutrients for body functions, and it also aims to maintain or recover a good nutritional status, decreasing the risk of complications, promoting quick recovery, and reducing length of stay at hospital, morbidity and mortality.^1-3^

Usually, there are two alternative administration routes for patients who are unable to ingest or digest food sufficiently to avoid malnourishment. Enteral nutrition is the process of provision of food to the gastrointestinal tract - typically in liquid form - by means of a nasogastric or orogastric feeding tube, or of a gastric or intestinal feeding tube placed by endoscopy or surgery. Parenteral nutrition aims to supply nutrients to a patient through a venous route, when it is not possible to provide the diet through the gastrointestinal tract, whether this is due to obstruction, difficulty in absorption, or inappropriate digestion of food.^1,2^ By and large, enteral nutrition is preferentially used because of the lower cost and risk of complications (especially infection).^4-6^

Analysis of the medical and hospital accounts, based on the activity of an analytic internal audit, can contribute towards the identification of fundamental indicators to subsidize the management of health insurance companies. One of these indicators is obtained by means of cost analysis, which contributes towards detecting the primary items of healthcare expenses. The main costs in health are generally related admissions to hospital (60% of costs) and treatments (10% of costs).^7^ Materials and medications, including the NT supplies, are the primary costs of hospitalization, accounting for 57.7%.^7^ On the other hand, it is also known that inpatients with malnutrition can increase the medical and hospital costs by 19 to 29%.^8,9^ There are few economic evaluation studies to subsidize the decision of physicians and healthcare managers when choosing the most cost-effective supplementary NT.^10,11^

## OBJECTIVE

The primary objective of this study was to perform an economic analysis of the enteral and parenteral nutritional therapy costs, according to the type of disease and outcome (survivors *versus* deaths).

## METHODS

A cross-sectional, documental, retrospective study was carried out, with a qualitative and quantitative strategy, during the period from January to December 2015, in a healthcare provider classified as self-governing, with 76,801 users of the city of Curitiba (PR).^12^

During the study period, ten hospital services linked to the healthcare provider and located within the same municipality, were qualified to administer enteral and parenteral NT. The study sample was obtained based on a random choice of hospital accounts of such services that used enteral and parenteral NT during 2015. This study excluded the hospital accounts of pediatric users.

The demand began with the admission of the user to one of the hospital services accredited by the healthcare provider. Whenever necessary, the user was evaluated by the Multiprofessional Nutritional Therapy Team (MNT) of the hospital service, composed of dietitian and a physician nutrition specialist. After analyzing the case and identifying the need of an enteral diet and parenteral diet, the MNT of the hospital requested from the healthcare provider authorization to begin therapy by filling out a form with rationale, user information and diagnosis, type of diet, quantity, and treatment period. The request was analyzed by the medical inspection of the healthcare provider, and the treatment was given pending on prior authorization. Only the diets recognized and approved by the National Health Surveillance Agency (ANVISA - *Agência Nacional de Vigilância Sanitária*) were authorized. Experimental treatments or diets with no national registration were not authorized. Only industrialized, not homemade, diets were used. Any change in the type of diet was made by means of a request of the hospital’s MNT to the healthcare provider and prior authorization of the medical inspection. The user’s NT was periodically evaluated by the medical audit team through analysis of the documents (medical records) and by an *in loco* visit.

Any information that could identify the user was excluded from the study. The data on sex, age, disease, type of diet, death, and costs were obtained from the information made available by the MNT of the hospitals to the medical audit of the healthcare provider in the requests for treatment and in the medical and hospital accounts analyzed.

The diseases were grouped by the investigators, according to users and hospital accounts, as follows: infection (regardless of the source), diabetes, cancer, cerebro-cardiovascular diseases (including stroke, heart diseases, thromboembolism, peripheral arterial occlusive disease), kidney disease (chronic or acute renal failure), chronic obstructive pulmonary disease (COPD), liver disease (cirrhosis, hepatitis, and dementia syndrome (especially Alzheimer’s disease). Each user or medical and hospital account could have registry of more than one disease.

Costs were calculated in national currency – *Reals* (R$) – based on the hospital accounts forwarded by the service providers to the healthcare provider, every 15 days. Additionally, the values were also converted into American dollar (US$), based on the mean exchange rate during the study period. Considering the exchange rate (for purchase) during 2015, the mean was R$ 3,34 (standard deviation – SD±R$ 0,43), ranging from R$ 2,57 to R$ 4,14.^13^

The costs were checked by using direct costs, that is, those that are clearly identified and quantified. The total account cost (TAC) was calculated by adding all the values spent with medical fees, procedures, levies, materials/supplies and medications, including the diets. The total diet cost (TDC) represents the sum of the values of the parenteral and enteral diet flasks used for each account, excluding medical fees and related materials/supplies (tube and catheter, among others). The TDC was subdivided into enteral diet costs (EDC) and parenteral diet costs (PDC), according to the type and feeding used in each account. Additionally, the cost indicators per day of hospitalization and per user, based on the value of the accounts, of the diets, and according to the modality of NT were used.

Each disease group was evaluated according to the number of accounts and users seen, as well as the cost indicators. Finally, the users who died and their respective accounts were compared to the surviving users during the study period as to sex, mean age, days of hospitalization, use of diet, and the cost indicators.

Study data were recorded and analyzed using a Microsoft Office Excel 2010^®^ software spreadsheet. Results of the quantitative variables were described as mean, median, and SD. Qualitative variables were demonstrated by frequencies and percentages.

## RESULTS

A total of 301 hospital accounts were analyzed regarding NT of 159 users linked to the healthcare provider under study. There were 208 accounts with only enteral diet, 32 accounts with only parenteral diet, and 61 accounts with both diets (concomitant use or not).

As to demographic profile, 88 users were males (55.4%). The mean age was 68.7 years (SD±11.7), median of 69, range of 35-92 years. The total length of stay for all accounts enrolled in the study was 3,084 days, with a mean of 10.24 (SD±3.69), median of 10, range of 1-26 days. The total number of diet days was 2,193, with a mean of 7.28 (SD±3.68), median of 7, ranging from 1 to 15 days.

The total cost of the 301 hospital accounts during the study period, including diets, medical fees, materials/supplies, and medications, was R$ 9.289.588,27 (US$ 2,781,313.85), in which R$ 58.425,08 (US$ 17,492.53) was per user (SD±R$ 63.652,38), median of R$ 35.773,55 (US$ 10,710.64), ranging from R$ 1.596,29 (US$ 477.93) to R$ 397.701,91 (US$ 119,072.42). Approximately 35.44% of the total value of the hospital accounts were with diet only (R$ 3.292.697,00; US$ 985,837.42), in which 59.82% were of the enteral modality (R$ 1.969.725,29; US$ 589,738.11) and 40.18% of the parenteral type (R$ 1.322.971,72; US$ 396,099.10). The TDC per user was R$ 20.708,79 (U$ 6,200.23), with SD±R$ 28.505,62, median of R$ 10.291,31 (US$ 3,081.23), varying from R$ 418,27 (US$ 125.23) to R$ 227.185,01 (US$ 68,019.46). The enteral TDC per user was R$ 13.131,50 (US$ 3,931.58), SD±R$ 16.345,49, median of R$ 8.210,36 (US$ 2,458.19), varying from R$ 418,27 (US$ 125.23) to R$ 110.426,38 (US$ 33,061.79). The total cost with parenteral diet per user was R$ 29.399,37 (US$ 8,802.20), SD± R$ 36.011,54, median of R$ 15.293,18 (US$ 4,578.79), varying between R$ 838,68 (US$ 251.10) and R$ 188.391,30 (US$ 56,404.58).

As to the disease group, the most frequent were infections (144 hospital accounts in reference to 83 users), cancers (83 hospital accounts from 46 users), and cerebro-cardiovascular diseases (71 hospital accounts from 45 users). The least frequent were liver diseases (eight hospital accounts in reference to four users) and diabetes (nine hospital accounts from six users), as per table 1.

**Table 1:** Demonstration of cost analysis in nutritional therapy per disease group

Disease group	Accounts	Users	Total account cost	Total diet cost	Enteral diet cost	Parenteral diet cost
Infection	144	83	R$ 4.651.935,91	R$ 1.481.243,56	R$ 1.046.075,95	R$ 435.167,61
			US$ 1,392,795.18	US$ 443,486.09	US$ 313,196.39	US$ 130,289.70
Diabetes	9	6	R$ 411.260,08	R$ 113.975,15	R$ 88.411,90	R$ 25.563,25
			US$ 123,131.76	US$ 34,124.29	US$ 26,470.62	US$ 7,653.66
Cancer	83	46	R$ 2.500.518,08	R$ 1.030.362,45	R$ 365.558,73	R$ 664.803,72
			US$ 748,658.10	US$ 308,491.75	US$ 109,448.72	US$ 199,043.02
Cerebro-cardiovascular disease	71	45	R$ 2.108.048,73	R$ 740.776,27	R$ 521.472,44	R$ 219.303,83
			US$ 631,152.31	US$ 221,789.30	US$ 156,129.47	US$ 65,659.82
Renal disease	35	21	R$ 1.114.337,32	R$ 358.043,12	R$ 253.732,44	R$ 104.310,68
			US$ 333,633.92	US$ 107,198.53	US$ 75,967.79	US$ 31,230.74
Chronic obstructive pulmonary diseases	45	17	R$ 1.501.344,09	R$ 439.783,04	R$ 422.973,15	R$ 16.809,89
			US$ 44,9504.21	US$ 131,671.56	US$ 126,638.66	US$ 5,032.90
Liver disease	8	4	R$ 271.783,61	R$ 92.008,45	R$ 79.231,99	R$ 12.776,46
			US$ 81,372.33	US$ 27,547.44	US$ 23,722.15	US$ 3,825.28
Dementia syndrome	20	13	R$ 437.886,73	R$ 202.870,79	R$ 109.196,70	R$ 93.674,09
			US$ 131,103.81	US$ 60,739.75	US$ 32,693.62	US$ 28,046.13

Considering the hospital TAC per disease group, three conditions are noteworthy - infections (R$ 4.651.935,91; US$ 1,392,795.18), cancers (R$ 2.500.518,08; US$ 748,658.10), and cerebro-cardiovascular diseases (R$ 2.108.048,73; US$ 631,152.31). The same disease groups also presented with the highest total diet costs, *i.e*., R$ 1.481.243,56 (US$ 443,486.09) for infections, R$ 1.030.362,45 (US$ 308,491.75) for cancers, and R$ 740.776,27 (US$ 221,789.30) for cerebro-cardiovascular diseases. The enteral diet presented with a higher total cost in infections (R$ 1.046.075,95; U$ 313,196.39), followed by the cerebro-cardiovascular diseases (R$ 521.472,44; US$ 156,129.47) and COPD (R$ 422.973,44; US$ 126,638.75). Whereas the parenteral diet had a higher total cost in cancers (R$ 664.803,72; US$ 199,043.02), followed by infections (R$ 435.167,61; US$ 130,289.70) and cerebro-cardiovascular diseases (R$ 219.303,83; US$ 65,659.82).

The highest ratio between the total diet cost and the hospital TAC was observed in dementia syndromes (46.32%) and cancers (41.20%), and the lowest ratio was in diabetes (27.71%) and COPD (29.29%). The cost ratio between the enteral diet and the total diet was greater in COPD (96.18%), in liver diseases (86.11%), and in diabetes (77.57%), whereas the cost ratio between the parenteral diet and total diet was greater in cancers (64.52%) and dementia syndromes (46.17%).

The cost of NT per day of hospital stay is demonstrated on table 2. One can observe the longer length of stay and greater period of NT use were in the group of infections (1,525 and 1,063, respectively), of cancers (779 and 562, respectively), and of cerebro-cardiovascular diseases (712 and 505, respectively). Additionally, liver diseases presented with the greatest number of days of NT use during hospital stay (the diet was used in 80% of total days of hospital admission), and diabetes showed the lowest number of days (50%). As to hospital TAC per day of hospitalization, liver diseases and cancers stood out (R$ 3.397,30 or US$ 1,017.15 and R$ 3.209,91 or US$ 961.05, respectively), and dementia syndromes presented the lowest values (R$ 2.419,26; US$ 724.32). As to TDC per day of hospitalization, the disease groups with the highest values were cancers (R$ 1.322,67; US$ 396.00), followed by liver diseases (R$ 1.150,11; US$ 344.34), and by dementia syndromes (R$ 1.120,83; US$ 335.57); the lowest values were found with diabetes (R$ 850,56; US$ 254.65). The highest costs with enteral diet per day of hospitalization were in liver diseases (R$ 990,40; US$ 296.52) and in COPD (R$ 822,90; US$ 246.37); whereas with parenteral diet, cancers stood out (R$ 853,41; US$ 255.51), as well as dementia syndromes (R$ 603,30; US$ 180.62). On the other hand, the lowest costs for enteral diet per day of hospitalization were in cancers (R$ 469,27; US$ 140.50) and in dementia syndromes (R$ 517,54; US$ 154.95); and with parenteral diet, it was in COPD (R$ 32,70; US$ 9.79) and liver diseases (R$ 159,71; US$ 47.81).

**Table 2:** Demonstration of cost analysis per day of hospitalization in nutritional therapy per disease groups

Disease group	Length of stay (days)	Diet use (days)	Total account cost per day of hospital stay	Total diet cost per day of hospital stay	Enteral diet cost per day of hospital stay	Parenteral diet cost per day of hospital stay
Infection	1,525	1,063	R$ 3.050,45	R$ 971,31	R$ 685,95	R$ 285,36
			US$ 913.30	US$ 290.81	US$ 205.37	US$ 85.43
Diabetes	134	67	R$ 3.069,11	R$ 850,56	R$ 659,79	R$ 190,77
			US$ 918.89	US$ 254.65	US$ 197.54	US$ 57.11
Cancer	779	562	R$ 3.209,91	R$ 1.322,67	R$ 469,27	R$ 853,41
			US$ 961.05	US$ 396.00	US$ 140.50	US$ 255.51
Cerebrocardiovascular disease	712	505	R$ 2.960,74	R$ 1.040,42	R$ 732,41	R$ 308,01
			US$ 886.44	US$ 311.50	US$ 219.28	US$ 92.21
Renal disease	359	259	R$ 3.104,00	R$ 997,33	R$ 706,78	R$ 290,56
			US$ 929.34	US$ 298.60	US$ 211.61	US$ 86.99
Chronic obstructive pulmonary disease	514	391	R$ 2.920,90	R$ 855,61	R$ 822,9	R$ 32,7
			US$ 874.52	US$ 256.17	US$ 246.37	US$ 9.79
Liver disease	80	64	R$ 3.397,30	R$ 1.150,11	R$ 990,4	R$ 159,71
			US$ 1,017.15	US$ 344.34	US$ 296.52	US$ 47.81
Dementia syndrome	181	129	R$ 2.419,26	R$ 1.120,83	R$ 517,54	R$ 603,3
			US$ 724.32	US$ 335.57	US$ 154.95	US$ 180.62

During the study period, the number of deaths was 59 users, 33 of them males, totaling up 134 hospital accounts. The mortality rate during the year was 37.10%, or 3.09% a month. The mean age of users who died was 71.08 years (SD±9.21), median of 70, range 51-90 years. In the study sample, the highest rates of mortality were found among users with COPD (58.82%), followed by diabetes (50%) and liver diseases (50%), kidney diseases (47.61%), cerebro-cardiovascular diseases (40%), infections (37.34%), cancers (34.78%), and dementia syndromes (23.07%).

Considering the 134 hospital accounts of the 59 users who died, the total cost was R$ 4.761.446,27 (US$ 1,425,582.71), accounting for 51.26% of total cost, *i.e*., R$ 80.702,48 (US$ 24,162.41) per user, with a SD±R$ 75.429,13, median R$ 50.646,07 (US$ 15,163.49), minimum of R$ 1.596,29 (US$ 477.93) and maximum of R$ 397.701,91 (US$ 119,072.42). In this group, the total diet cost was 32.81% of total value of the hospital accounts and 47.45% of total diet value (R$ 1.562.539,37; US$ 467,826.15). The EDC was R$ 784.504,70 (US$ 234,881.64), *i.e*., 39.38% of total diet cost, with a mean of R$ 13.525,94 (US$ 4,049.68) per user (SD±R$ 13.773,28), and a median of R$ 8.774,51 (US$ 2,627.09), varying from R$ 774,55 (US$ 231.90) to R$ 49.836,96 (US$ 14,921.24). Regarding parenteral diet, the cost was R$ 778.034,69 (US$ 232,944.51), 58.81% of the total, with mean of R$ 38.901,73 (US$ 11,647.22) per user (SD±R$ 46.690,75), median of R$ 22.286,62 (US$ 6,672.64), varying from R$ 838,68 (US$ 251.10) to R$ 188.391,30 (US$ 56,404.58), as shown in table 3.

**Table 3:** Demonstration of cost analysis in nutritional therapy, as per outcome (deaths *versus* survivors)

	Deaths	%	Survivors	%	Total
Number of users	59	37.1	100	62.9	159
Male	33	37.5	55	62.5	88
Female	26	36.6	45	63.4	71
Mean age	71.08	-	67.42	-	68.77
Number of accounts	134	44.5	167	55.5	301
Length of stay (days)	1,432	46.4	1,652	53.6	3,084
Diet use (days)	966	44.3	1,215	55.7	2,181
Total account cost	R$ 4.761.446,27	51.26	R$ 4.528.142,00	48.74	R$ 9.289.588,27
	US$ 1,425,582.71		US$ 1,355,731.13		US$ 2,781,313.85
Total diet cost	R$ 1.562.539,37	47.45	R$ 1.730.157,64	52.55	R$ 3.292.697,01
	US$ 467,826.15		US$ 518,011.26		US$ 985,837.42
Enteral diet cost	R$ 784.504,68	39.83	R$ 1.185.220,61	60.17	R$ 1.969.725,29
	US$ 234,881.64		US$ 354,856.47		US$ 589,738.11
Parenteral diet cost	R$ 778.034,69	58.81	R$ 544.937,03	41.19	R$ 1.322.971,72
	US$ 232,944.51		US$ 163,154.79		US$ 396,099.31

On the other hand, 167 hospital accounts related to other 100 surviving users were identified, 55 of them males. In this group, the TAC was R$ 4.528.142,00 (US$ 1,355,731.14), accounting for 48.74% of total cost, with a mean of R$ 45.281,42 (US$ 13,557.31) per user (SD±R$ 51.601,27), median of R$ 27.153,14 (US$ 8,129.68), minimum of R$ 1.727,25 (US$ 517.14) and maximum of R$ 326.899,09 (US$ 97,873.98). In this group, the total cost with diet was 38.20% relative to the total value of accounts, and 52.55% of total value in diets (R$ 1.730.157,64; US$ 518,011.27). The EDC was R$ 1.185.221,00 (US$ 354,856.59), that is, 60.17% of total diet cost, with a mean of R$ 12.882,83 (US$ 3,857.13) per user (SD±R$ 17.845,89), median of R$ 8.140,35 (US$ 2,437.23), range of R$ 418,27 (US$ 125.23) to R$ 110.426,40 (US$ 33,061.80). In the parenteral diet, the cost was R$ 544.937,00 (US$ 163,154.79), that is, 41.19% of total in diet, with a mean of R$ 21.797,48 (US$ 6,526.19) per user (SD±R$ 22.720,62), and a median of R$ 13.926,77 (US$ 4,169.69), varying from R$ 4.088,48 (US$ 1,224.10) to R$ 105.224,70 (US$ 31,504.40), as per table 3.

## DISCUSSION

In Brazil, some studies demonstrated an institutional mortality rate with a median of 2.6% a month, varying between zero to 15.7%.^14^ In hospitals with acutely ill patients, the mortality rate varies from 3.4 to 5.3%; in chronic patients under palliative care, it is 18.2%; and in teaching hospitals, it is 4.7 to 6.8%.^14^ As to length of hospital stay, the mean time was 4 to 5 days, ranging from 0.2 to 18.6 days.^14^ Comparing with the data found in this study, one can conclude that the NT was used more frequently in older inpatients, with a mortality rate greater than the national median, and with a mean rate of hospital stay relatively higher than that found in private hospitals in Brazil. We should point out that both the mortality rate and the length of stay depend on several factors, including the clinical profile of the patients seen, procedures offered, specialties available, and size and level of complexity of each service. The clinical condition usually observed in patients with NT may justify the greater mortality rate and longer hospital stay.

As to the relation between NT and diseases, we noted that infections and chronic non-communicable diseases (CNCD) were the primary conditions requiring enteral and parenteral diets. Additionally, most hospital accounts in users with infection (approximately 60% of them) presented with some related chronic disease. The profile of diseases identified in the study sample is consistent with the main causes of hospital admissions, paid by healthcare insurance providers, according to data from the *Agência Nacional de Saúde Suplementar* (ANS) [National Agency for Healthcare Provision through Insurance Companies].^12^ The most prevalent diseases (infections, cancers, and cerebro-cardiovascular diseases) also presented with a higher TDC. On the other hand, when we analyzed the total cost per day of hospitalization, we observed that the value was very similar among different diseases.

The method of choice for nutritional supplementation is the oral route, and when this is not possible, the enteral route is preferentially used because of its lower cost and lower risk of complications.^4-6^ Thus, it was expected that the higher NT costs would be in the enteral mode. Cancers present with the highest cost per day in terms of diet, especially due to parenteral NT. This result can be justified taking into consideration the gastrointestinal oncologic diseases submitted to surgical treatment that used the parenteral route for nutritional supplementation. The dementia syndromes, despite a lower cost per day of hospitalization, displayed a relatively high total diet cost per day, predominantly due to the parenteral diet. Based on the data analyzed, it was not possible to justify the reason for the costs in parenteral diet in the dementia syndromes.

Usually, certain disease groups have indications of special diets, such as diabetes, COPD, and liver diseases.^15^ Frequently, this group of special diets stands out because it represents a relatively higher cost than the other types of diet.^15^ In this case, the diabetes group showed the lowest cost in diet cost per day of hospitalization. However, one needs to consider that the same group also presented with the lowest ratio between days of diet use and hospitalization days, which could have justified the result found. COPD and liver diseases stood out for using NT for the highest number of days of hospitalization and at the lowest PDC.

Based on this sample, therefore, we recommend monitoring the NT costs in the group of cancers and dementia syndromes, according to the criteria of use, and with cost-benefit and cost-effectiveness ratios of the diets in each case. The standardization of specialized diets and the negotiation of values of the hospital services can contribute towards the reduction of costs in some diseases, such as COPD, diabetes, and liver and kidney diseases. Finally, the preventive actions against chronic diseases are important and can contribute towards the reduction of costs in hospital admissions and use of hospital diets in the long run.

In this study, the older users progressed to death with greater frequency. As was expected, the mortality and morbidity of the geriatric patients were greater than among the younger patients, due to the low functional reserves of organs and systems, and metabolic, biochemical, immunologic, and nutritional abnormalities.^16^ In the present sample, we observed that both the hospital TAC and the TDC per user were greater in the group of those who died (1.78 and 1.53 time greater, respectively). As to the type of diet, the cost of the parenteral modality per user also was noteworthy in the group that died (2.42 times higher). There are studies showing that malnutrition can increase costs in healthcare.^8,9^ Nevertheless, the cost-effectiveness studies on NT use are still scarce and inconclusive.^10,11^ Considering that the resources in healthcare are limited, the decision for choice of treatment should take into account, whenever possible, the clinical protocols, evidence-based medicine, the gain in quality of life, and survival. Cost-effectiveness and cost-utility studies are necessary to identify the groups that benefit the most from enteral and parenteral NT, guaranteeing the best results and the best application of the resources available.^5,17-20^

As to the limitations of this study, one should take into account that the results portray the reality of a specific segment paid by healthcare insurance in Brazil, in the form of self-management and regional coverage. In 2015, the participation of this modality of healthcare provider in the Brazilian healthcare market was 11%.^12^ Considering the total number of users, we can classify it as of medium size, *i.e*., between 20,000 and 100,000 users, which represents 23% of healthcare insurance providers in the country.^12^ We also point out that the results were obtained from the records in hospital accounts and depend on the correct registry of the information. Usually the recording of values is in conformity with the service rendered and paid for by the healthcare provider. However, the diagnostic information might be incomplete, reflecting only the primary disease, without considering the comorbidities.

Regarding the outcome analysis (deaths and survivors), we should consider that the groups are not homogeneous and are not necessarily exposed to the same risk. Finally, there was no control group, *i.e*., hospital accounts of users under similar conditions and without NT, with the objective of comparing the costs and results.

## CONCLUSION

Infections, cancers, and cerebro-cardiovascular diseases were the morbidities that used nutritional therapy with greatest frequency and that presented with the highest total diet cost. Cancers presented with the highest cost in terms of diet per day of hospitalization. Parenteral nutritional therapy exceeded the cost of the enteral modality in cancers and dementia syndromes. The total hospital account costs per user as well as the total diet cost per user were greater in the group in which patients died, especially in the parenteral therapy modality. We recommend new economic analysis studies in nutritional therapy in other healthcare providers, as well as in the public healthcare system, to compare with the results found in this study.
